# Atomoxetine/Methylphenidate Effects on Social Play Behavior

**DOI:** 10.15844/pedneurbriefs-29-2-1

**Published:** 2015-02

**Authors:** J. Gordon Millichap

**Affiliations:** 1 Division of Neurology, Ann & Robert H. Lurie Children's Hospital of Chicago, Chicago, IL; 2 Departments of Pediatrics and Neurology, Northwestern University Feinberg School of Medicine, Chicago, IL

**Keywords:** Amygdala, Habenula, Methylphenidate, Noradrenaline, Prefrontal Cortex, Social Play Behavior

## Abstract

Researchers at Utrecht University, The Netherlands, and University “Roma Tre,” Rome, Italy, studied the neural substrates of the previously identified social play-suppressant effects of methylphenidate (MPH) and atomoxetine, drugs widely used for the treatment of attention-deficit hyperactivity disorder (ADHD).

Researchers at Utrecht University, The Netherlands, and University “Roma Tre,” Rome, Italy, studied the neural substrates of the previously identified social play-suppressant effects of methylphenidate (MPH) and atomoxetine, drugs widely used for the treatment of attention-deficit hyperactivity disorder (ADHD) [1, 2]. During adolescence, rats display a characteristic highly vigorous form of social behavior, termed social play behavior. MPH was infused into prefrontal and orbitofrontal cortical regions in rats and into several subcortical limbic areas that are implicated in cognition, emotion and social play. Infusion of MPH into the anterior cingulate cortex, infralimbic cortex, amygdala, and habenula inhibited social play, but not exploratory behavior or locomotor activity. Infusion of the noradrenaline reuptake inhibitor atomoxetine into these same regions also reduced social play. MPH administration into the prelimbic, medial/ventral orbitofrontal and ventrolateral orbitofrontal cortex, mediodorsal thalamus, or nucleus accumbens shell was ineffective. The inhibitory effects of MPH and atomoxetine on social play are mediated through a noradrenergic mechanism and via a network of prefrontal and limbic subcortical regions implicated in cognitive control and emotional/social behavior. [1]

COMMENTARY. The inhibition of social play observed in animals following brain infusion with MPH or atomoxetine [1] is consistent with a similar effect reported in children prescribed MPH for ADHD. In hyperactive boys, aged 7-12, observed as “leaders” for groups of younger children, MPH had a general dampening effect on social behavior, significantly reducing social engagement and increasing dysphoria relative to placebo [3]. Aversive social behaviors, a so-called “zombi” appearance and dysphoria are not uncommon in children with ADHD treated with larger doses of MPH [4].

Atomoxetine, used to treat ADHD as an alternative to stimulant medication, is occasionally associated with suicide-related behavior [4]. A meta-analysis of 14 trials found the frequency of suicidal ideation was 0.37% versus 0% in the placebo group; no patient committed suicide. Although uncommon, suicidal ideation is significantly more frequent in ADHD patients treated with atomoxetine than controls [5]. Close monitoring for the occurrence of suicidal behavior is recommended when treatment with atomoxetine is begun in children with ADHD. Inhibition of social behavior, an adverse event frequently overlooked in children receiving methylphenidate and eloquently demonstrated in these animal experiments, should receive greater attention clinically.
